# Increased expression of fatty acid and ABC transporters enhances seed oil production in camelina

**DOI:** 10.1186/s13068-021-01899-w

**Published:** 2021-02-27

**Authors:** Guangqin Cai, Geliang Wang, Sang-Chul Kim, Jianwu Li, Yongming Zhou, Xuemin Wang

**Affiliations:** 1 Key Laboratory of Biology and Genetic Improvement of Oil Crops, Ministry of Agriculture and Rural Affairs, Oil Crop Research Institute, Chinese Academy of Agricultural Sciences, Wuhan, 430062 Hubei China; 2 National Key Laboratory of Crop Genetic Improvement, Huazhong Agricultural University, Wuhan, 430070 Hubei China; 3 Department of Biology, University of Missouri, St. Louis, MO 63121 USA; 4 Donald Danforth Plant Science Center, St. Louis, MO 63132 USA

**Keywords:** Camelina, Lipid metabolism, Oil production, Seed weight, Transporters

## Abstract

**Background:**

Lipid transporters play an essential role in lipid delivery and distribution, but their influence on seed oil production in oilseed crops is not well studied.

**Results:**

Here, we examined the effect of two lipid transporters, *FAX1* (*fatty acid export1*) and *ABCA9* (*ATP-binding cassette transporter subfamily A9*) on oil production and lipid metabolism in the oilseed plant *Camelina sativa*. Overexpression (OE) of *FAX1* and *ABCA9* increased seed weight and size, with *FAX1*-OEs and *ABCA9*-OEs increasing seed length and width, respectively, whereas *FAX1*/*ABCA9*-OEs increasing both. *FAX1-*OE and *ABCA9-*OE displayed additive effects on seed oil content and seed yield. Also, OE of *FAX1* and *ABCA9* affected membrane lipid composition in developing pods, especially on phosphatidylcholine, phosphatidylethanolamine, and phosphatidylglycerol. The expression of some genes involved in seed oil synthesis, such as *DGAT2*, *PDAT1*, and *LEC1*, was increased in developing seeds of *FAX1*- and/or *ABCA9*-OEs.

**Conclusion:**

These results indicate that increased expression of *FAX1* and *ABCA9* can potentially be applied to improving camelina oil production.

## Background

Fatty acids (FAs) are the major and essential component of membrane lipids and important energy stores for metabolism and cellular energy homeostasis [1, 2]. In addition, FAs participate in many regulatory processes in organismal growth, development, and stress responses. In plants, FAs are synthesized in plastids and exported out of plastids and to endoplasmic reticulum (ER) for elongation, desaturation, and other embellishments [3, 4]. One transporter, FAX1 (fatty acid export 1), a membrane protein in chloroplast inner envelopes, was identified to transport FAs out of chloroplasts in *Arabidopsis thaliana* [5]. Overexpression (OE) of *FAX1* led to an increase in ER-derived lipids and a decrease in several plastid-produced lipids in flowers and leaves [5]. *FAX1* overexpression also increased the biomass production and seed oil content in Arabidopsis [5, 6]. After acyl-CoA transported to cytosol, it bound to acyl-CoA-binding proteins (ACBPs), which might contribute to drive plastid FA-export [7].

FA associated with ER is involved in the biosynthesis of triacylglycerol (TAG). An ER-localized ATP-binding cassette (ABC) transporter subfamily A, ABCA9, was described to transport FA/acyl-CoA to the ER [8]. Developing seeds of *ABCA9*-knockout mutant (*abca9*) incorporated less ^14^C-oleoyl-CoA into TAG compared with WT seeds. OE of *ABCA9* enhanced TAG deposition by up to 40%, with enlarged seeds, larger embryo, more densely packed with oil bodies [8]. When *ABCA9* was overexpressed, the seed size and seed oil content were increased, and *abca9* had opposite effects [8]. However, the function of FAX1 and ABCA9 and influence on seed oil accumulation in oilseed crops remain to be tested.

*Camelina sativa*, an archaic oilseed crop had cultivation more than 3000 years [9, 10]. Camelina was an important oilseed crop in Europe and Asia for centuries [11, 12], but its cultivation declined in the past century and was replaced by higher-yielding crops, such as rapeseed (*Brassica napus*) [9, 13]. Camelina has several advantages as an oilseed crop. Camelina seed oil contains a high level of unsaturated FAs (> 90%), with the level of polyunsaturated α-linolenic acid being 30–40% of the total oil [14–16]. In addition, camelina seed meals contain a low level of glucosinolates, toxic for feed use [9, 16]. Furthermore, camelina is a low-input crop with a low requirement for water and nutrients, and it is resistant to common Brassicaceae pests and pathogens and adaptable to hostile environmental conditions [11, 17, 18]. Moreover, camelina has a short life cycle with 85–100 days from seeds to seeds, and it is easy to transform genetically [19, 20]. In recent years, biotechnological manipulations of one or multiple genes have been employed to modify camelina seed oil compositions. Those include the production of fish oil-like levels (> 12%) of polyunsaturated fatty acids to achieve a high ω3/ω6 ratio [21–25], oleyl oleate wax esters [26], acetyl glyceride oils [27], poly-3-hydroxybutyrate [28], ω-7 monounsaturated fatty acids [29], cyclopropane fatty acids [30], and oleic acid levels from 16% to over 50% [31], as well as decreased levels of α-linolenic acid levels [32] and of C20–C24 very long-chain fatty acids to less than 2% of total fatty acids [33]. However, low seed and oil yield relative to other oil crop, such as canola, are the major concerns for camelina production. In this study, we overexpressed the two Arabidopsis transporters, *FAX1* and *ABCA9*, in camelina to test their functions on oil accumulation, yield and other agronomic traits, and lipid metabolism in developing pods.

## Results

### Overexpression of *FAX1* and *ABCA9* in camelina

**Fig. 1:**
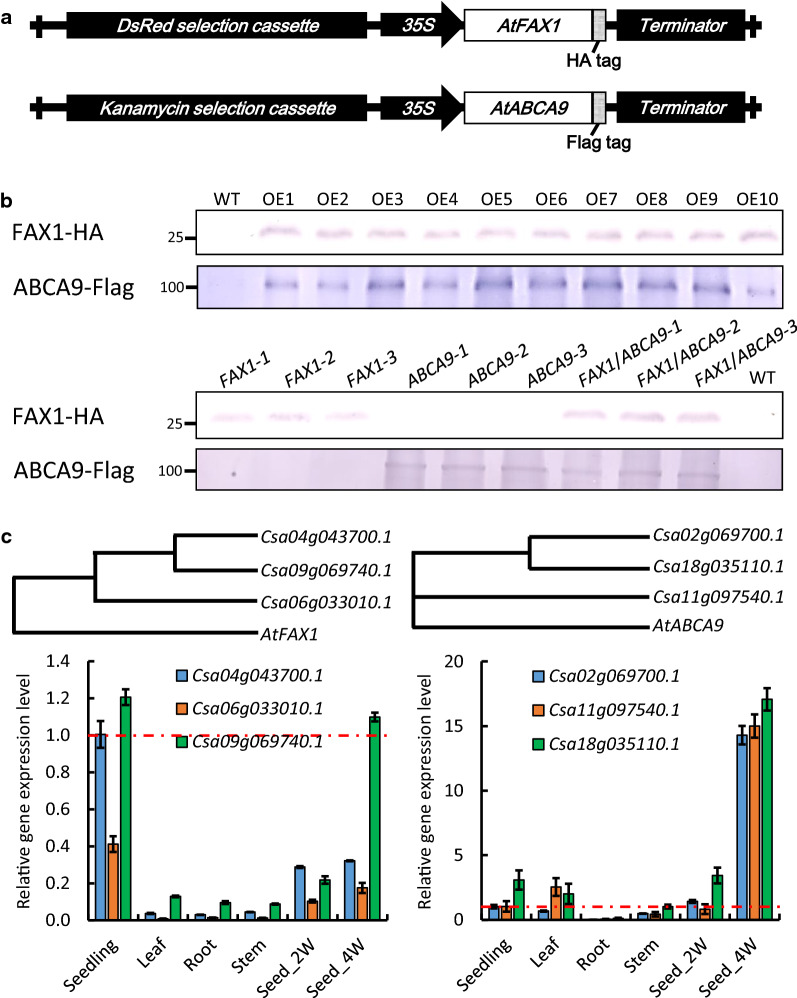
Overexpression of *AtFAX1* and *AtABCA9* in camelina.** a** The overexpression (OE) constructs of *AtFAX1* and *AtABCA9*. **b** Immunoblotting of HA-tagged FAX1 and Flag-tagged ABCA9 in *AtFAX1-*OE, *AtABCA9-*OE, and *AtFAX1*/*ABCA9-*OE camelina leaves. Total proteins (10 µg/lane) were extracted from leaves of 3-week-old plants, separated by 10% SDS-PAGE, and transferred to a polyvinylidene difluoride membrane. The membrane was blotted with anti-HA or anti-Flag antibody conjugated with alkaline phosphatase. Lanes OE1 through OE10 represent different transgenic lines harboring the FAX1-HA or ABCA9-Flag OE construct. Numbers on the left of each panel mark protein molecular mass standards in kilodaltons. **c** The phylogenetic tree of *FAX1* (*left*) and *ABCA9* (*right*) in camelina, and their expression pattern in seedlings, leaves, roots, stems, and 2- and 4-week (W) old developing seeds. *ACT2* (*actin2*) was used as the internal standard for cDNA input adjustment. The expression level is relative to the value of *Csa04g043700.1* for *FAX1* and *Csa02g069700.1* for *ABCA9* in seedlings (red dashed line). Values are means ± SE (*n* = 3)

To investigate the function of *FAX1* and *ABCA9* in camelina, we overexpressed Arabidopsis *FAX1* and *ABCA9* genomic DNAs under the control of the cauliflower mosaic virus (CaMV)-35S promoter (Fig. 1a). The constitutive promoter was used because constitutive expression of these genes were reported to promote plant growth in Arabidopsis in addition to lipid production [5, 8]. FAX1 and ABCA9 were fused with a HA-tag and a Flag-tag at the C-terminus, respectively. The production of FAX1-HA and ABCA9-Flag in camelina was confirmed by immunoblotting using anti-HA and anti-Flag antibodies, respectively (Fig. 1b, *upper* panel). The homozygous OE plants producing FAX1-HA and ABCA9-Flag were identified. In addition, we generated camelina lines overexpressing both *FAX1* and *ABCA9* by transforming the *FAX1-*HA construct into homozygous *ABCA9*-OE plants*.* The production of FAX1-HA and ABCA9-Flag in *FAX1*/*ABCA9* single and double OEs was confirmed by immunoblotting (Fig. 1b, *lower* panel). Camelina has 3 homeologs each of *FAX1* and *ABCA9* (Fig. 1c), and in WT camelina the transcript levels of the three *FAX1s* were relatively high in seedlings and developing seeds, but low in leaves, roots, and stems (Fig. 1c, *left* panel). The transcript levels of the three *ABCA9* were relatively high in 4-week-old developing seeds (Fig. 1c, *right* panel).

### *FAX1* and *ABCA9* increase seed and oil yield

**Fig. 2:**
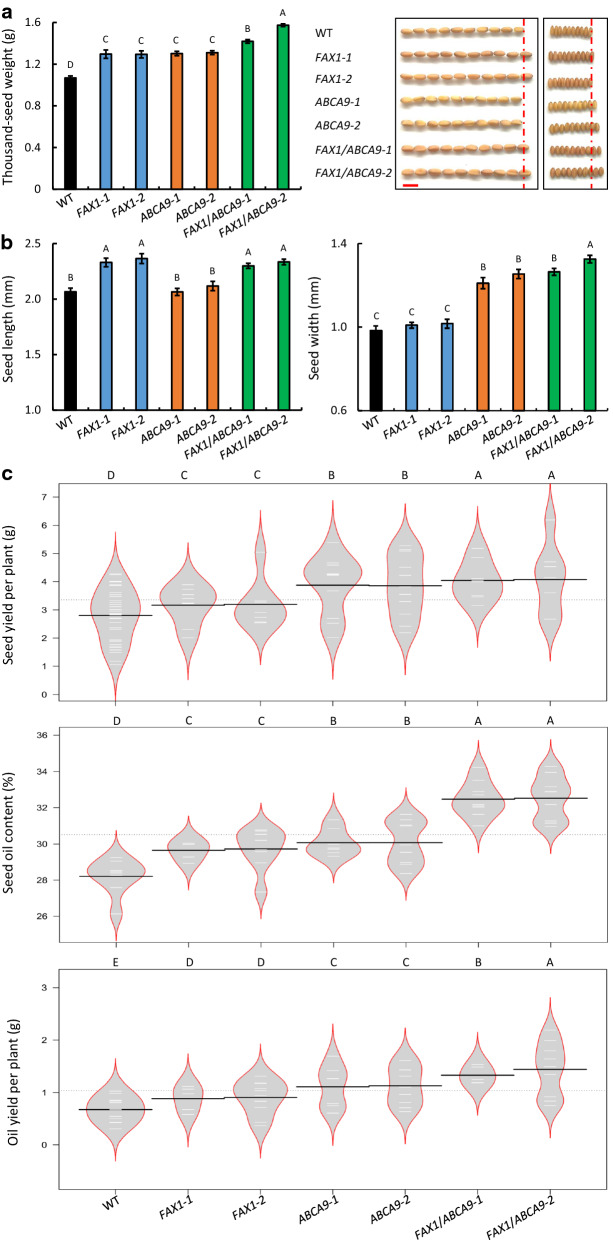
Effect of *AtFAX1*- and *AtABCA9*-OEs on seed weight, seed size, seed oil content, and oil yield.** a** Thousand-seed weight and seed morphology of camelina dry seeds. Values are means ± SE (n = 12). Bar = 3 mm. **b** Seed length and width of camelina dry seeds. Values are means ± SE (*n* = 30). **c** Seed yield per plant (g), Seed oil content (w/w), and oil yield per plant (g) of different camelina lines. *AtFAX1*- and *AtABCA9*-single and double OEs were grown side by side together with WT. Seeds from whole plant were harvested and dried at room temperature for at least one month before measurement. Seed oil content was measured by gas chromatography, and calculated based on the internal standard (C17:0) peak area. Seeds from each plant were measured with 3 technical replicates, and the mean values are represented here as the seed oil content of each plant. Black lines show the averages, white lines represent individual data points (*n* = 18), and polygons represent the estimated density of the data. Homozygous, T3-generation OE lines were used for this and subsequent characterization. Capital letters indicate a significant difference (*P* < 0.01) based on *Duncan*-test

*FAX1*-OEs, *ABCA9*-OEs, *FAX1*/*ABCA9*-OEs, and WT plants were grown side by side to determine the effect of *FAX1* and *ABCA9* on camelina growth and production. The OE lines had similar flowering time, plant height, and branch number as WT (Additional file 1: Fig. S1). However, the thousand-seed weights (TSWs) of *FAX1*-OEs and *ABCA9*-OEs were 21% and 22% higher on average than that of WT, respectively, and *FAX1*/*ABCA9*-OEs were 40% higher on average than that of WT (Fig. 2a). Interestingly, *FAX1*-OEs and *ABCA9*-OEs had distinctive effect on seed length and seed width. *FAX1*-OEs increased seed length by 14%, whereas *ABCA9*-OEs increased seed width by 25%, while the seed length and width of *FAX1*/*ABCA9*-OEs were increase by 13% and 32%, respectively (Fig. 2a, b). The seed width of *FAX1*-OEs and seed length of *ABCA9*-OEs were similar with those of WT (Fig. 2b). The above results indicate that *FAX1* and *ABCA9* increase seed length and width, respectively, to increase seed weight.

To determine the effect of *FAX1* and *ABCA9* on oil production in camelina, we grew the single and double OE plants together with WT side by side with multiple replicates (*n* = 18). Compared to WT, the seed yield per plant on average was 14% higher for *FAX1*-OE, 38% higher for *ABCA9*-OE, and 45% higher for *FAX1*/*ABCA9*-OEs (Fig. 2c). Moreover, the average oil content of WT seeds was 28%, whereas that *FAX1*-OE and *ABCA9*-OE seeds were 29% and 30%, respectively, and that of *FAX1*/*ABCA9*-OE seed was 32% (Fig. 2c). The average increase of seed oil content over WT was 4% for *FAX1*-OE, 6% for *ABCA9*-OE, and 13% for *FAX1*/*ABCA9*-OE seeds. The results indicate that *FAX1* and *ABCA9* increase seed oil content and seed yield simultaneously and these two genes had an additive effect on seed oil content and seed yield, leading a substantial improvement on overall oil yield. Combining the increases in seed oil content and seed yield, the oil production per plant of *FAX1*-OEs and *ABCA9*-OEs was 22% and 55% higher than that of WT, respectively, whereas that of *FAX1*/*ABCA9*-OEs was 75% higher than that of WT (Fig. 2c).

The *FAX1*-OE and *ABCA9*-OE seeds displayed altered FA composition from WT seeds. Compared with that of WT seeds, the level of C16:0 of *FAX1*-OEs and *ABCA9*-OEs was decreased by 6% and 4%, respectively, whereas that of *FAX1*/*ABCA9*-OEs was decreased by 14% (Additional file 2: Fig. S2). Conversely, the level of C18:0 was increased by 5% for *FAX1*-OEs and *ABCA9*-OEs, and 12% for *FAX1*/*ABCA9*-OEs. The level of C18:1 was increased by 13% and 16% for *FAX1*-OEs and *ABCA9*-OEs, respectively, and 29% for *FAX1*/*ABCA9*-OEs. The level of C18:2 was increased by 6% and 7% for *FAX1*-OEs and *ABCA9*-OEs, respectively, and 10% for *FAX1*/*ABCA9*-OEs whereas that of C18:3 was decreased by 4% for *FAX1*-OEs and *ABCA9*-OEs and 10% for *FAX1*/*ABCA9*-OEs comparing to that of WT. In addition, the level of C20:1 was decreased by 4% and 11% in *FAX1*-OE and *ABCA9*-OE seeds, and 10% in *FAX1*/*ABCA9*-OE seeds (Additional file 2: Fig. S2). The above results indicate that increased *FAX1* and *ABCA9* expressions positively affect the level of C18:0, C18:1 and C18:2, but negatively affect the level of C16:0, C18:3 and C20:1.

To test the seed performance, we monitored seed germination rates of the above lines. The seed germination rate at 12 h after imbibition of *FAX1* and *ABCA9* single and double OEs was slightly faster than that of WT while the final seed germination rate at 60 h after imbibition of all the lines tested was about 99% (Additional file 3: Fig. S3). In addition, we examined the vegetative growth of the above lines by growing the *FAX1*-OEs, *ABCA9*-OEs, and *FAX1*/*ABCA9*-OEs with WT side by side. The plant size appeared to be bigger in the single and double OE plants compared with WT (Additional file 4: Fig. S4a). The overground fresh weight of 3-week-old *FAX1*-OE and *ABCA9*-OE plants was 24% and 27% higher on average than that of WT, respectively, whereas that of *FAX1*/*ABCA9*-OE was 57% higher on average than WT (Additional file 4: Fig. S4b). Also, *FAX1*-OEs had one more, *ABCA9*-OEs had two more, *FAX1*/*ABCA9*-OEs had 2–3 more leaves of 3-week-old plants compared with WT (Additional file 4: Fig. S4b).

### *FAX1* and *ABCA9* affect membrane glycerolipid composition

**Fig. 3:**
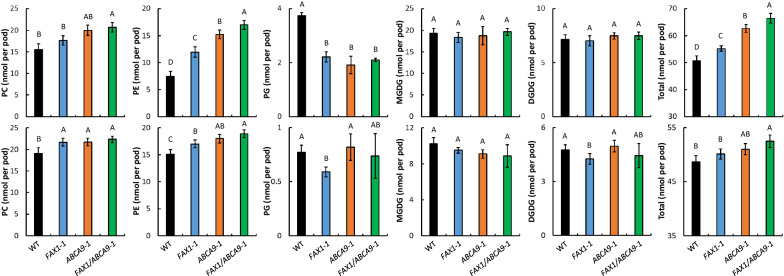
Alterations of membrane glycerolipid levels (nmol per pod) in developing pods of *AtFAX1*- and *AtABCA9*-OEs. 2-week (*upper* panels) and 4-week (*lower* panels) old developing pods were sampled for lipid profiling by ESI–MS/MS. The total lipid levels referred to the total amount of major phospholipids (PC, PE, and PG) and galactolipids (MGDG and DGDG) measured. Values are means ± SE with 5 biological replicates. Capital letters on the top of each panel indicate a significant difference (*P* < 0.01) based on *Duncan*-test. PC: phosphatidylcholine; PE: phosphatidylethanolamine; PG: phosphatidylglycerol; MGDG: monogalactosyldiacylglycerol; and DGDG: digalactosyldiacylglycerol

We further examined the effect of *FAX1*-OE and *ABCA9*-OE on membrane glycerolipid composition in seed pods containing developing seeds. Whole pods, instead of dissected seeds, were used because seed dissections would lead to wounding and activation of lipolytic activities. Total lipids were extracted from developing pods of 2 and 4 weeks after flowering from WT, and *FAX1*-1, *ABCA9*-1, and *FAX1*/*ABCA9*-1 OE lines, and analyzed using electrospray ionization tandem mass spectrometry (ESI–MS/MS). In 2-week-old pods (WOPs), which were considered as the outburst stage of lipid synthesis characterized by rapid lipid synthesis and oil production [34], compared to WT, the amount of phosphatidylcholine (PC) was increased 13%, 28%, and 33% in *FAX1*-1, *ABCA9*-1, and *FAX1*/*ABCA9*-1, respectively (Fig. 3). The amount of phosphatidylethanolamine (PE) was increased 60%, 104%, and 127% in *FAX1*-1, *ABCA9*-1, and *FAX1*/*ABCA9*-1. The total membrane glycerolipid level was 9%, 24%, and 31% higher in *FAX1*-1, *ABCA9*-1, and *FAX1*/*ABCA9*-1 than WT at the early stage of developing pods. However, the amount of phosphatidylglycerol (PG) was decreased approximately 45% in all three OE lines, and the level of monogalactosyldiacylglycerol (MGDG) and digalactosyldiacylglycerol (DGDG) had no obvious changes (Fig. 3). When the lipid data were calculated as mol% of total lipids analyzed, PE in *FAX1*-1, *ABCA9*-1, and *FAX1*/*ABCA9*-1 was 6, 12, and 13 mol% higher, respectively, than that of WT. However, the major plastidic lipids, phosphatidylglycerol (PG), MGDG, and DGDG were all lower in three OE lines than WT (Additional file 5: Fig. S5). Phosphatidic acid (PA) constituted less than 0.2 mol% in the developing pod of all lines tested and the PA mol% in *FAX1*-OE, *ABCA9*-OE, and *FAX1*/*ABCA9*-OE lines was all lower than that of WT (Additional file 5: Fig. S5).

In 4-WOPs, which was considered as the plateau stage of lipid synthesis [34], the level of PC in *FAX1*-1, *ABCA9*-1, and *FAX1*/*ABCA9*-1 was 14%, 14%, and 17% higher than that of WT (Fig. 3). The level of PE in *FAX1*-1, *ABCA9*-1, and *FAX1*/*ABCA9*-1 was 12%, 19%, and 25% higher than that of WT. The total membrane glycerolipid content in *FAX1*-1, *ABCA9*-1, and *FAX1*/*ABCA9*-1 was 3%, 5%, and 8% higher than that of WT (Fig. 3). However, the level of major plastidic lipids, PG and DGDG was decreased in *FAX1*-1 whereas was comparable among WT, *ABCA9*-1 and *FAX1*/*ABCA9*-1 at this stage (Fig. 3). PC in *FAX1*-1, *ABCA9*-1, and *FAX1*/*ABCA9*-1 was 2, 3, and 4 mol% higher than WT whereas mol% of MGDG and DGDG in the three OE lines was slightly lower than that of WT. The mol% of PE and PG was comparable between WT and OE lines at this stage (Additional file 5: Fig. S5). Also, the PA mol% was lower all OE lines than WT (Additional file 5: Fig. S5).

**Fig. 4:**
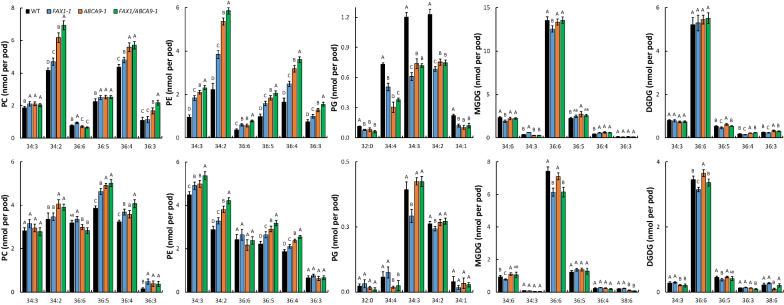
Alterations of PC, PE, PG, MGDG, and DGDG species (nmol per pod) in developing pods of *AtFAX1*- and *AtABCA9*-OEs. 2-week (*upper* panels) and 4-week (*lower* panels) old developing pods were sampled for lipid profiling by ESI–MS/MS. The species levels were measured by internal standards. Values are means ± SE with 5 biological replicates. Capital letters on the top of each panel indicate a significant difference (*P* < 0.01) based on *Duncan*-test

The major PC species (34:3, 34:2, 36:5, and 36:4 PC) in *FAX1*-1, *ABCA9*-1, and *FAX1*/*ABCA9*-1 were increased in 2-WOPs, and 36:5, 36:4, and 36:3 PC were increased in 4-WOPs compared with those of WT (Fig. 4). Similarly, the major PE species (34:3, 34:2, 36:6, 36:5, 36:4, and 36:3 PE) of *FAX1*-1, *ABCA9*-1, and *FAX1*/*ABCA9*-1 were all increased in 2-WOPs, and only 34:3, 34:2, 36:5, and 36:4 PE were increased in 4-WOPs compared to those of WT (Fig. 4). Also, the effect of *FAX1* and *ABCA9* exhibited additive effect, especially on 34:2 and 36:4 PC, and 34:3, 34:2, 36:5, and 36:4 PE (Fig. 4). In contrast, the major PG species (32:0, 34:4, 34:3, 34:2, and 34:1 PG) of *FAX1*-1, *ABCA9*-1, and *FAX1*/*ABCA9*-1 were all decreased compared to those of WT in 2-WOPs, while only 34:3 PG was decreased in *FAX1*-1, and 34:4 PG decreased in *ABCA9*-1, and *FAX1*/*ABCA9*-1 in 4-WOPs (Fig. 4). Although the total amount of MGDG was similar between WT and OE lines (Fig. 3), the level of some MGDG species, especially in *FAX1*-1, was different from that of WT. For instance, two major MGDG spices, 34:6 and 36:6 MGDG, in *FAX1*-1 was decreased both in 2- and 4-WOPs compared with WT (Fig. 4). For DGDG, only 36:5 DGDG in 2-WOPs, and 36:6 and 36:5 DGDG in 4-WOPs of *FAX1*-1 were lower than WT (Fig. 4). Similarly, the mol% of 34:2 PC of *ABCA9*-1 and *FAX1*/*ABCA9*-1 in 2-WOPs, and 36:5 PC of *ABCA9*-1 and *FAX1*/*ABCA9*-1 in 4-WOPs were higher than that of WT (Additional file 6: Fig. S6); the mol% of 34:2 PE of *ABCA9*-1 and *FAX1*/*ABCA9*-1 was higher than WT both in 2- and 4-WOPs; whereas the mol% of 34:2 PG of *FAX1*-1 was lower than WT; the major MGDG species 36:6 was lower in *FAX1*-1 and *FAX1*/*ABCA9*-1; and the major DGDG species 36:6 was lower in *FAX1*-1 in 4-WOPs (Additional file 6: Fig. S6). The above lipid data indicate that overexpression of *FAX1* and *ABCA9* also affect membrane lipid composition in developing pods, and the effect differs at different developmental stage.

### *FAX1* and *ABCA9* alter the expression of genes in oil production in seeds

**Fig. 5:**
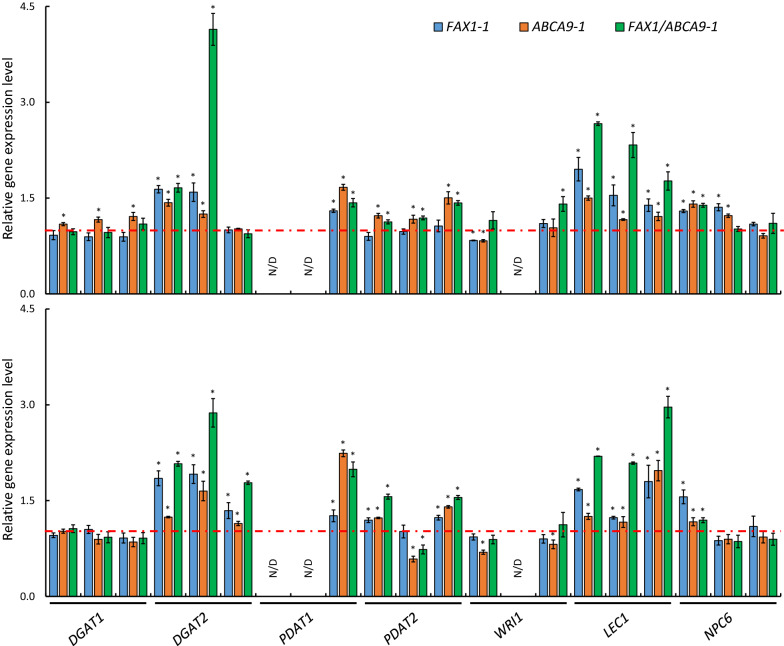
Effect of *AtFAX1*- and *AtABCA9*-OEs on the expression of genes involved in oil accumulation. Samples were collected with 2-week (*upper* panel) and 4-week (*lower* panel) old developing seeds for RNA extraction and real-time PCR. *ACT2* (*actin2*) was used as the internal standard for cDNA input adjustment. The three bars of each genes indicated the three copies of each gene in camelina. Values are the relative expression level compared with WT (red dashed line) and are means ± SE (*n* = 3). *Significant difference (*P* < 0.05) based on Student’s* t* test compared with WT. N/D: not detected. *DGAT*: *Acyl-CoA: diacylglycerol acyltransferase*; *PDAT*: *phospholipid: diacylglycerol acyltransferase*; *WRI1*: *wrinkled 1*; *LEC1*: *leaf cotyledon 1*; and *NPC6*: *nonspecific phospholipase C 6*

To gain insights into the enhancing oil production, we compared the transcript level of genes that are involved in oil accumulation in developing seeds of the *FAX1-* and *ABCA9-*OE lines with WT. The transcript level of *AtFAX1* in 2- and 4-week-old developing seeds (WODS) was high in *FAX1*-OE and *FAX1*/*ABCA9*-OE lines, but was not detected in *ABCA9*-OE lines, as expected. Similarly, the level of *AtABCA9* was high in *ABCA9*-OE and *FAX1*/*ABCA9*-OE lines, but was not detected in *FAX1-*OE lines (Additional file 7: Fig. S7). The transcript level of three *DGAT1s* (*Acyl-CoA: diacylglycerol acyltransferase 1*) was higher in *ABCA9*-1, and slightly lower in *FAX1*-1 in 2-WODS whereas that of two *DGAT2s* was higher in 2-WODS, and all three *DGAT2* were higher in 4-WODS in all OE lines than WT (Fig. 5). The transcript of one *PDAT1* (*phospholipid: diacylglycerol acyltransferase 1*) was detected, and its level was higher than that of WT in all OE lines at both stages. The level of three *PDAT2s* transcripts was higher in *ABCA9*-1 and *FAX1*/*ABCA9*-1 in 2-WODS, whereas that of two *PDAT2s* transcripts was higher in three OE lines but the other *PDAT2* was lower in *ABCA9*-1 and *FAX1*/*ABCA9*-1 in 4-WODS. The transcript level of one *WRI1* was lower in *FAX1*-1 and *ABCA9*-1 whereas another *WRI1* was higher in *FAX1*/*ABCA9*-1 in 2-WODS (Fig. 5). At 4-WODS, the level of two *WRI1s* was lower in *ABCA9*-1 than WT. The transcript levels of *LEC1* (*leaf cotyledon 1*) homeologs were higher in 2- and 4-WODS in all OE lines than WT. One *NPC6* (*nonspecific phospholipase C 6*) displayed a higher level in *FAX1*- and *ABCA9*-OEs in 2- and 4-WODS, and another had a higher level in *FAX1*-1 and *ABCA9*-1 in 2-WODS (Fig. 5).

## Discussion

In this study, we show that OE of two Arabidopsis transporters, *FAX1* and *ABCA9*, in camelina improves seed and oil production in the emerging oil crop. While the positive effect of *FAX1*-OE and *ABCA9*-OE in camelina is generally consistent with prior reports on the two genes being overexpressed individually in Arabidopsis [5, 6], the effect of simultaneously overexpressing the two transporters was previously unknown. A key new finding of the present study is that co-OE of *FAX1* and *ABCA9* has an additive effect on enhancing seed and oil production. In addition, our comparison of the two gene effects side by side in camelina led to other new findings. One is that *FAX1* and *ABCA9* exhibited different effects on seed size; whereas *FAX1*-OE increased seed length, *ABCA9* -OE increased seed width. Several genes that affect differently only seed length or width were reported in rice [35–42], but those genes, such as those encoding a heterotrimeric G protein subunit and an E3 ubiquitin ligase, share no apparent common function with the two transporters. It would be of great interests in future studies to probe how the two transporter genes have such distinctive effects in camelina seed shape.

Another finding from the comparative analysis is that OE of the two transporters has different effects on lipid composition. *FAX1*-OE was lower than WT and *ABCA9*-OE in both the amount (nmol per pod) and mol% of plastidic lipids, such as 34:3- and 34:2-PG, 34:6- and 36:6-MGDG in 2- and 4-WOPs. In comparison, *ABCA9*-OE was higher than WT and *FAX1*-OE in the amount and mol% of extra-plastidic lipids, including 34:2-PC, and 34:2-PE in 2-WOPs, and 34:2- and 36:5-PC, and 34:2-, 36:5-, and 36:4-PE in 4-WOPs. FAX1 was localized at the inner envelope of the chloroplasts transporting FAs out of chloroplasts [5], whereas ABCA9 was associated with ER and proposed to transport FAs to ER [8].The decrease of plastidic lipids in *FAX1*-OE may result from an enhanced transport of FAs from plastids to cytosol/ER by FAX1, whereas the increase of extra-plastidic lipids in *ABCA9*-OE may result from enhanced FA transport to ER by ABCA9 for glycerolipid synthesis. The distinctive effects of FAX1 and ABCA9 on FA transport and lipid metabolism may help to explain the additive enhancements of *FAX1* and *ABCA9* on seed and oil production.

Furthermore, *FAX1-* and *ABCA9-*OE developing camelina seeds also displayed increased level of expression of specific genes related to TAG production, such as *DGAT2*, *PDAT1*, and *LEC1*. Those increases may result from an increase in supply of fatty acids and metabolic demand as the overall activity for lipid production and/or more substrates are available for those enzymes. *ABCA9*-OE and *FAX1*-OE exhibited different impacts on the expression of these genes. The expression level of *DGAT1* and *PDAT2* in 2-WODS was increased in *ABCA9*-1 and/or *FAX1*/*ABCA9*-1 but not in *FAX1*-1 compared to that in WT, which may explain the reason of a higher seed oil content in *ABCA9*-OEs than that in *FAX1*-OEs. It should be noted that all the observations of the present study are based on laboratory conditions and application of the promising results awaits further testing in field conditions, including the response of the transporter-altered lines under field growth environments.

## Conclusion

Here, we show that OE of *FAX1* and *ABCA9* increased seed weight, size, and oil production in camelina and co-OE of the two genes has an additive effect on the enhancement. *FAX1*- and *ABCA9*-OEs had different effects on seed length and seed width. *FAX1*-OEs and *ABCA9*-OEs increase seed length and width, respectively, whereas co-OE of *FAX1* and *ABCA9* increases both seed length and width. The results indicate that simultaneous OE of *FAX1* and *ABCA9* may potentially be applied to improving camelina oil production.

## Methods

### Plant materials and growth conditions

To overexpress Arabidopsis *ABCA9* transporter in camelina, the genomic sequence of *ABCA9* (AT5G61730) was amplified by PCR using Col-0 Arabidopsis genomic DNA as a template and by forward primer with *KpnI* site and reverse primer with *PacI* site. The C-terminal Flag tag was fused upstream of the terminator manually by adding its coding sequence to the reverse primer. Transgenic T1 plants were selected on a medium containing 50 mg/L kanamycin. The putative transgenic seedlings were transferred to soil and leaves were collected for PCR confirmation of the presence of *AtABCA9*. The PCR reaction condition was pre-incubated at 94 °C for 4 min, 35 cycles of 94 °C for 30 s, 58 °C for 30 s, and 72 °C for 1 min, and final extension at 72 °C for 10 min.

To overexpress Arabidopsis *FAX1* transporter in camelina, the genomic sequence of *FAX1* (AT3G57280) was amplified by PCR using Col-0 Arabidopsis genomic DNA as a template and by forward primer with *EcoRI* site and reverse primer with *SmaI* site. The C-terminal HA tag was fused upstream of the terminator manually by adding its coding sequence to the reverse primer. Putative *FAX1-*OE seeds were first identified by selecting red seeds under green flashlight with a red-light filter. Plants derived from putative transformed seeds were further verified by PCR, using the cloning primers and PCR condition as described above.

The details for gene cloning, plant transformation, putative transgenic plants identification were performed as described previously [43, 44]. Camelina plants were grown in greenhouse at 21 °C with approximately 16 h light (566 μmol/m^2^/s).

Homozygous lines of T3 OE lines were used to compare plant growth and yield traits among these OE lines and WT. We used 2.5-gallon pots with BM7-35% soil (Berger) and each pot had 4 plants with one as WT as control in greenhouse. There were 18 biological replicates with a completely randomized block design for each transgenic line. The greenhouse condition and plants managements were as described previously [43, 44]. Measurements of flowering time, plant height, branch number, thousand-seed weight, and plant yield per plant were performed as described [45]. All mother plants of the investigated lines were grown in the same condition and harvested at the same time, and seeds were stored under the same environment.

### Phylogenetic analysis

The coding sequences of *AtFAX1* and *AtABCA9* were used as queries to search for homologous genes in the camelina reference genome [12] using BLASTn program with an E-value of 1E-50 and an identity of 50% set as thresholds. The phylogenetic tree was drawn by Phylodendron (http://iubio.bio.indiana.edu/treeapp/treeprint-sample1.html).

### Immunoblotting and transcript analysis

Total protein extraction and immunoblotting were performed as described previously [44]. Protein concentrations were measured using the Bradford assay (Bio-Rad, 500–0205). RNA extraction, real-time PCR analysis, and semi-quantitative RT (reverse transcription)-PCR of transcript levels were performed as described previously [46, 47]. Total RNA was extracted from 2- and 4-week old developing seeds. Camelina *ACT2* (*Csa19g026200.1*) were used as internal standard and for cDNA input adjustment. All primers used in RT-PCR and real-time PCR are listed in Additional file 8: Table S1.

### Seed oil content and fatty acid composition analyses

Seed oil content and FA composition were determined as described previously [43, 44]. Fatty acid methyl esters (FAMEs) from TAG were identified by comparing their retention times with known standards. The FA composition was calculated as mol %.

### Lipid extraction and profiling

Polar lipids were extracted and analyzed by ESI–MS/MS based on a method described previously [44, 48]. The mass spectrometry data for lipids were processed using the software Analyst 1.5.1.

### Accession numbers

Sequence data from this article can be found in the following database under the accession numbers: Arabidopsis Genome Initiative database: *FAX1*, AT3G57280; and *ABCA9*, AT5G61730. *Camelina sativa* Genome Resources (http://www.camelinadb.ca/): *CsACT2*, Csa19g026200.1; *CsFAX1*: Csa09g069740.1, Csa06g033010.1, Csa04g043700.1; *CsABCA9*: Csa02g069700.1, Csa18g035110.1, Csa11g097540.1; *CsDGAT1*: Csa01g042590.1, Csa19g056370.1, Csa15g084220.1; *CsDGAT2*: Csa04g037310.1, Csa09g058550.1, Csa06g025650.1; *CsPDAT1*: Csa13g016300.1, Csa08g005560.1, Csa20g019000.1; *CsPDAT2*: Csa04g024660.1, Csa06g018480.1, Csa09g035780.1; *CsWRI1*: Csa06g028810.1, Csa09g064030.1, Csa04g040400.1; *CsLEC1*: Csa17g028800.1, Csa03g025850.1, Csa14g027200.1; and *CsNPC6*: Csa09g050690.1, Csa06g022410.1, Csa04g033750.1.

## Supplementary Information

**Additional file 1: Figure S1.** Effect of *AtFAX1*- and *AtABCA9*-OEs on other major agronomic traits.

**Additional file 2: Figure S2.** Effect of *AtFAX1*- and *AtABCA9*-OEs on seed fatty acid composition.

**Additional file 3: Figure S3.** Seed germination rate of *AtFAX1*- and *AtABCA9*-OEs.

**Additional file 4: Figure S4.** Effect of *AtFAX1*- and *AtABCA9*-OEs on vegetative tissues.

**Additional file 5: Figure S5.** Alterations of membrane glycerolipid levels (mol%) in developing pods of *AtFAX1*- and *AtABCA9*-OEs.

**Additional file 6: Figure S6.** Alterations of PC, PE, PG, MGDG, and DGDG species (mol%) in developing pods of *AtFAX1*- and *AtABCA9*-OEs.

**Additional file 7: Figure S7.** Gene expression level of *AtFAX1* and *AtABCA9* in developing seeds of camelina OE lines.

**Additional file 8: Table S1.** Primers (5′ to 3′) used for cloning and RT-PCR.

## Data Availability

All data generated or analyzed during this study are included in the article and its additional files.

## Abbreviations

FA: Fatty acid
ABC: ATP-binding cassette
FAX: Fatty acid export
TAG: Triacylglycerol
PC: Phosphatidylcholine
PE: Phosphatidylethanolamine
PG: Phosphatidylglycerol
PA: Phosphatidic acid
MGDG: Monogalactosyldiacylglycerol
DGDG: Digalactosyldiacylglycerol
