# Supplementary material for: Increased expression of fatty acid and ABC transporters enhances seed oil production in camelina
Source: Biotechnol Biofuels. 2021 Feb 27;14:49. doi: 10.1186/s13068-021-01899-w (PMC7913393; doi:10.1186/s13068-021-01899-w)
Supplement: Supplementary file 1 — Additional file 1: Figure S1. Effect of AtFAX1- and AtABCA9-OEs on other major agronomic traits. [file 13068_2021_1899_MOESM1_ESM.pptx]

## Slide 1
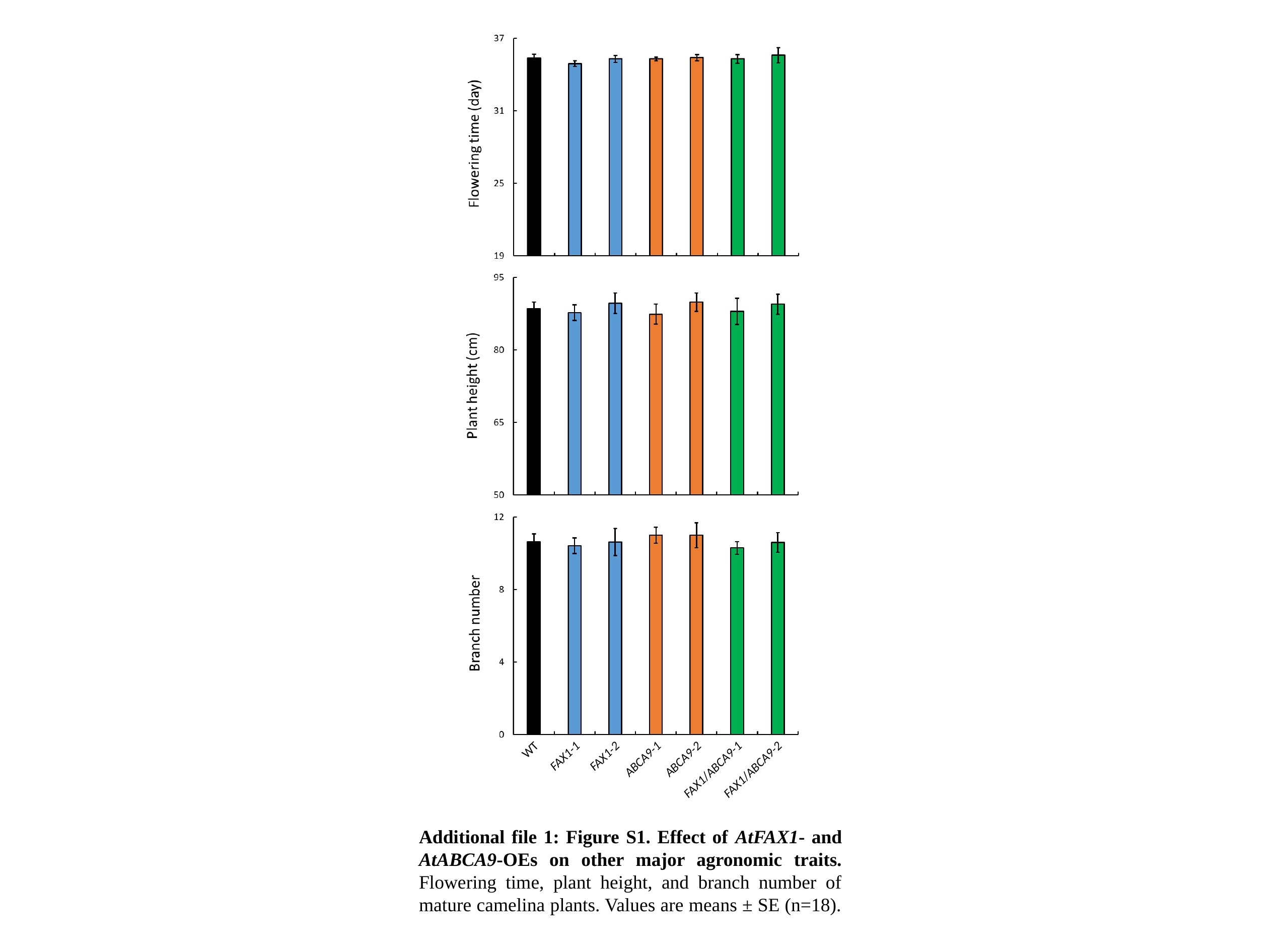

WT
FAX1-2
FAX1-1
ABCA9-2
ABCA9-1
FAX1/ABCA9-2
FAX1/ABCA9-1
Additional file 1: Figure S1. Effect of AtFAX1- and AtABCA9-OEs on other major agronomic traits. Flowering time, plant height, and branch number of mature camelina plants. Values are means ± SE (n=18).
