# Supplementary material for: Increased expression of fatty acid and ABC transporters enhances seed oil production in camelina
Source: Biotechnol Biofuels. 2021 Feb 27;14:49. doi: 10.1186/s13068-021-01899-w (PMC7913393; doi:10.1186/s13068-021-01899-w)
Supplement: Supplementary file 2 — Additional file 2: Figure S2. Effect of AtFAX1- and AtABCA9-OEs on seed fatty acid composition. [file 13068_2021_1899_MOESM2_ESM.pptx]

## Slide 1
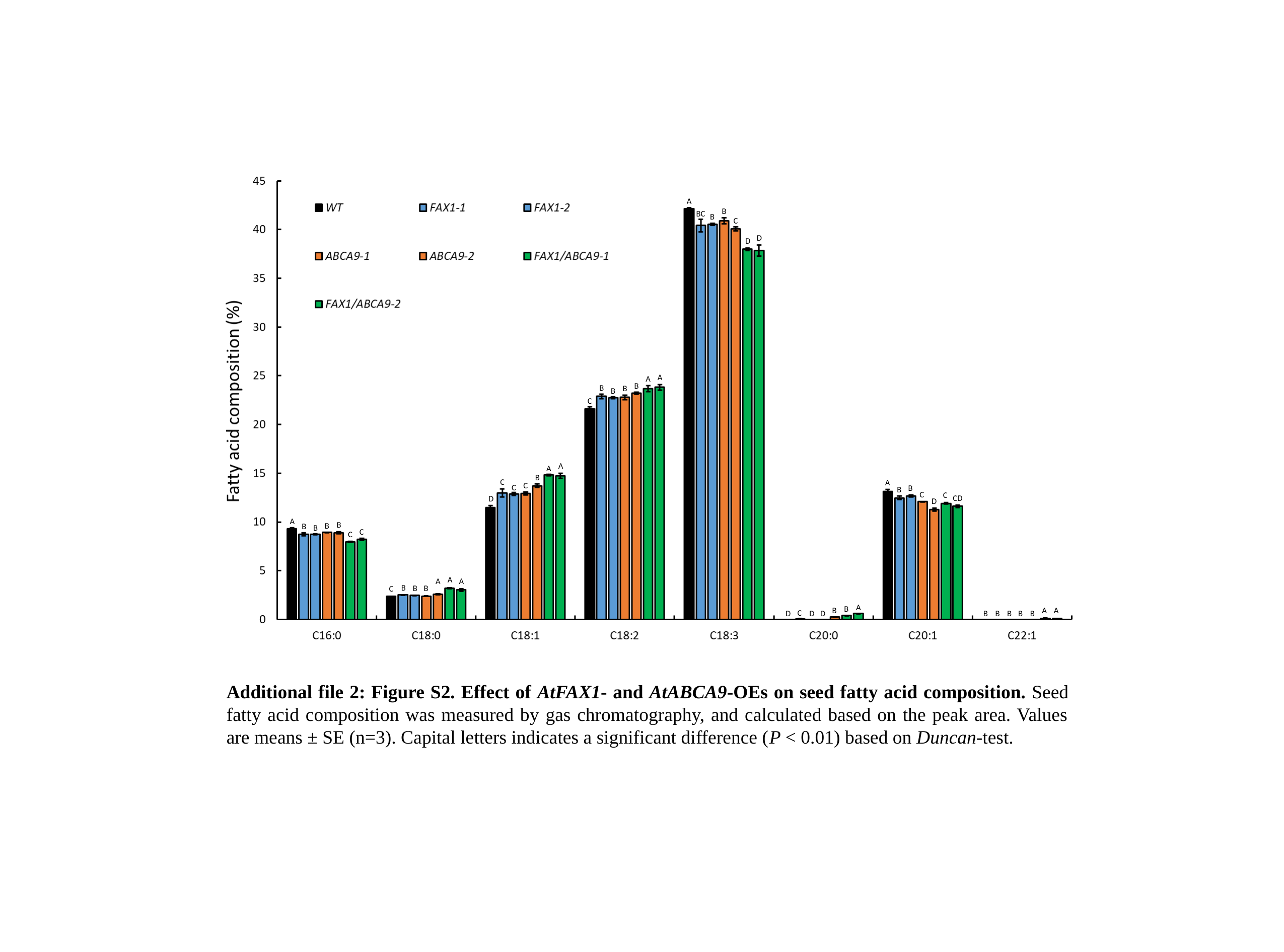

A
B
BC
B
C
D
D
A
A
B
B
B
B
C
A
A
B
C
A
C
C
B
B
C
C
CD
D
D
A
B
B
B
B
C
C
A
A
A
B
B
B
C
A
B
B
A
A
C
D
B
B
B
D
B
B
D
Additional file 2: Figure S2. Effect of AtFAX1- and AtABCA9-OEs on seed fatty acid composition. Seed fatty acid composition was measured by gas chromatography, and calculated based on the peak area. Values are means ± SE (n=3). Capital letters indicates a significant difference (P < 0.01) based on Duncan-test.
