# Supplementary material for: Increased expression of fatty acid and ABC transporters enhances seed oil production in camelina
Source: Biotechnol Biofuels. 2021 Feb 27;14:49. doi: 10.1186/s13068-021-01899-w (PMC7913393; doi:10.1186/s13068-021-01899-w)
Supplement: Supplementary file 3 — Additional file 3: Figure S3. Seed germination rate of AtFAX1- and AtABCA9-OEs. [file 13068_2021_1899_MOESM3_ESM.pptx]

## Slide 1
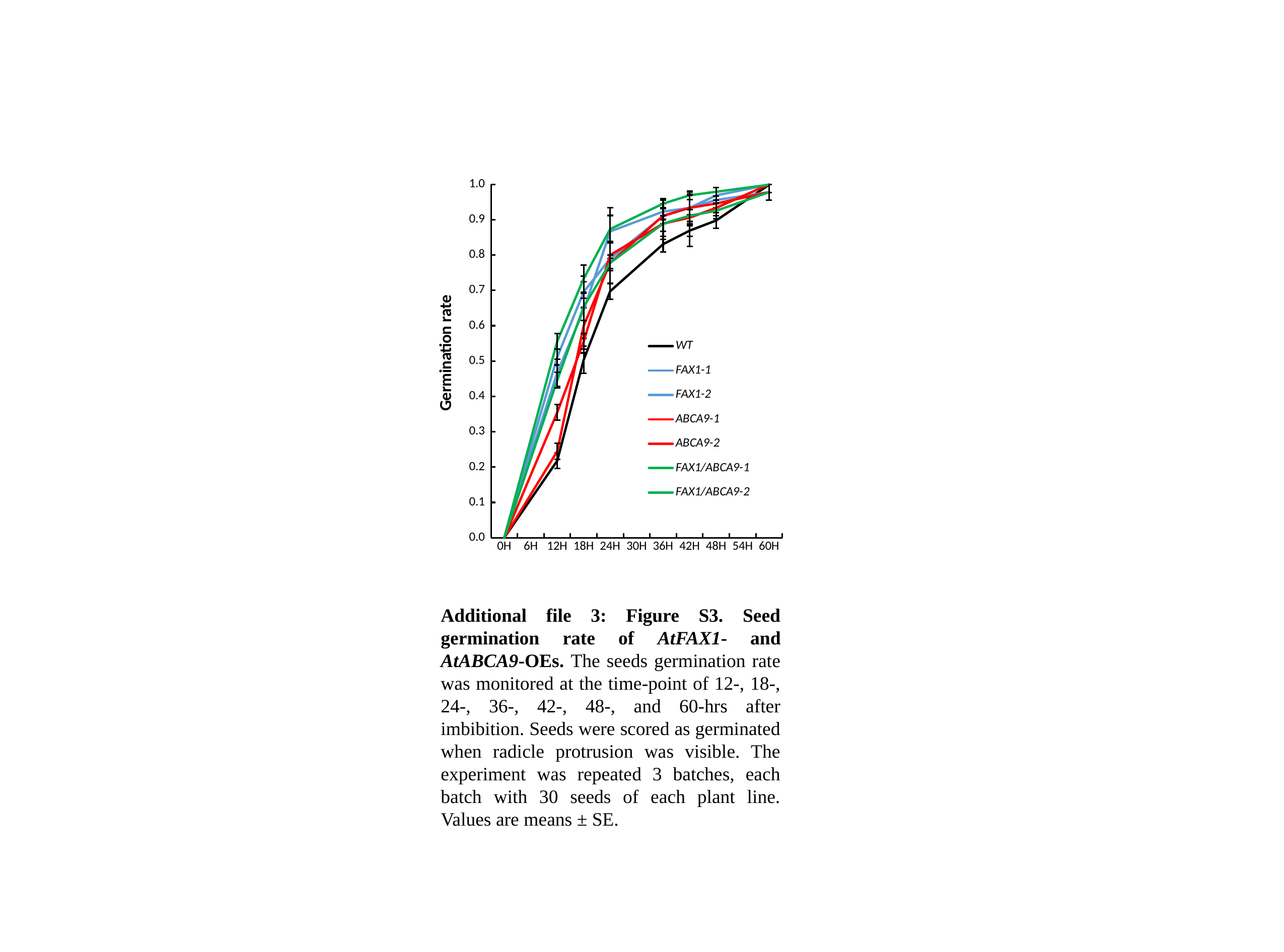

### Chart
| Category | WT | FAX1-1 | FAX1-2 | ABCA9-1 | ABCA9-2 | FAX1/ABCA9-1 | FAX1/ABCA9-2 |
|---|---|---|---|---|---|---|---|
| 0H | 0.0 | 0.0 | 0.0 | 0.0 | 0.0 | 0.0 | 0.0 |
| 6H | 0.1088888888888885 | 0.25555555555555554 | 0.2333333333333333 | 0.12222222222222223 | 0.177222222222222 | 0.27777777777777773 | 0.2228415555555555 |
| 12H | 0.217777777777777 | 0.5111111111111111 | 0.4666666666666666 | 0.24444444444444446 | 0.354444444444444 | 0.5555555555555555 | 0.445683111111111 |
| 18H | 0.503333333333333 | 0.695555555555555 | 0.644444444444444 | 0.6 | 0.5555555555555555 | 0.733333333333333 | 0.653333333333333 |
| 24H | 0.697511111111111 | 0.7895678 | 0.8666666666666667 | 0.7777777777777777 | 0.7999999999999999 | 0.873333333333333 | 0.7777777777777777 |
| 30H | 0.7640777777777774 | 0.8498589000000001 | 0.8947222222222219 | 0.8444444444444443 | 0.8444444444444444 | 0.9093611111111104 | 0.8333333333333333 |
| 36H | 0.830644444444444 | 0.91015 | 0.922777777777777 | 0.9111111111111111 | 0.888888888888889 | 0.945388888888888 | 0.888888888888889 |
| 42H | 0.86888888888888 | 0.93333 | 0.9333333333333332 | 0.9333333333333332 | 0.905555555555555 | 0.9688888888888 | 0.9111 |
| 48H | 0.897555555555555 | 0.9687 | 0.9555555555555556 | 0.94555 | 0.9333333333333332 | 0.978888888888888 | 0.925111111111111 |
| 54H | 0.9482171777777775 | 0.9837777777777775 | 0.9666666666666668 | 0.9616638888888889 | 0.9666666666666666 | 0.988888888888888 | 0.9514444444444444 |
| 60H | 0.9988788 | 0.998855555555555 | 0.9777777777777779 | 0.9777777777777779 | 1.0 | 0.998888888888888 | 0.9777777777777779 |Additional file 3: Figure S3. Seed germination rate of AtFAX1- and AtABCA9-OEs. The seeds germination rate was monitored at the time-point of 12-, 18-, 24-, 36-, 42-, 48-, and 60-hrs after imbibition. Seeds were scored as germinated when radicle protrusion was visible. The experiment was repeated 3 batches, each batch with 30 seeds of each plant line. Values are means ± SE.
