# Supplementary material for: Increased expression of fatty acid and ABC transporters enhances seed oil production in camelina
Source: Biotechnol Biofuels. 2021 Feb 27;14:49. doi: 10.1186/s13068-021-01899-w (PMC7913393; doi:10.1186/s13068-021-01899-w)
Supplement: Supplementary file 4 — Additional file 4: Figure S4. Effect of AtFAX1- and AtABCA9-OEs on vegetative tissues. [file 13068_2021_1899_MOESM4_ESM.pptx]

## Slide 1
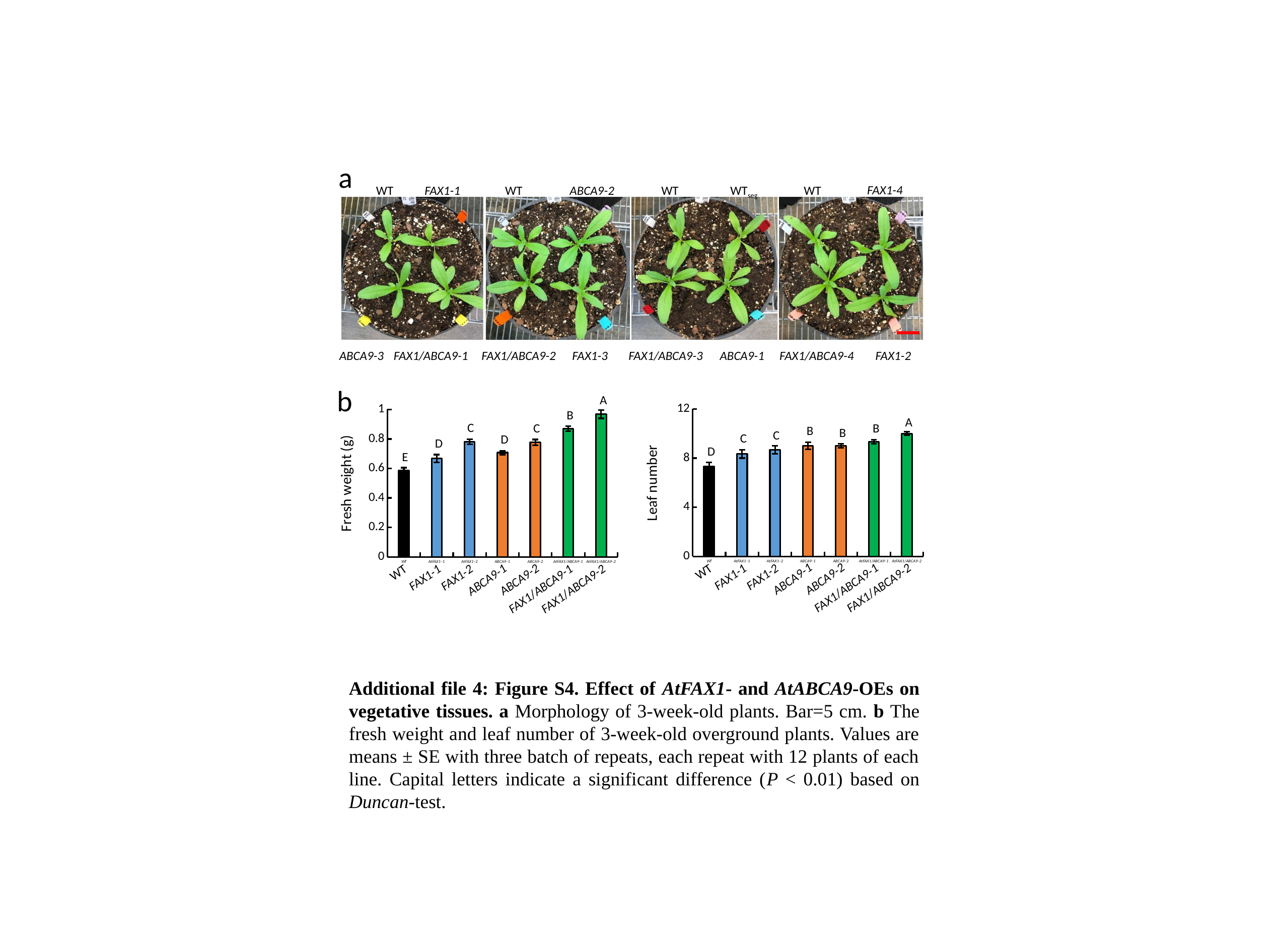

a
FAX1-4
WT
ABCA9-2
WT
WTseg
WT
WT
FAX1-1
FAX1-2
FAX1/ABCA9-4
FAX1/ABCA9-1
FAX1/ABCA9-2
FAX1-3
FAX1/ABCA9-3
ABCA9-1
ABCA9-3
b
A
### Chart
| Category | |
|---|---|
| WT | 7.333333333333333 |
| AtFAX1-1 | 8.333333333333334 |
| AtFAX1-2 | 8.666666666666666 |
| ABCA9-1 | 9.0 |
| ABCA9-2 | 9.0 |
| AtFAX1/ABCA9-1 | 9.333 |
| AtFAX1/ABCA9-2 | 10.0 |
### Chart
| Category | |
|---|---|
| WT | 0.5852 |
| AtFAX1-1 | 0.66698 |
| AtFAX1-2 | 0.7819875000000001 |
| ABCA9-1 | 0.7076333333333333 |
| ABCA9-2 | 0.7776333333333333 |
| AtFAX1/ABCA9-1 | 0.869875 |
| AtFAX1/ABCA9-2 | 0.9677666666666668 |B
A
C
B
C
B
B
C
C
D
D
D
E
WT
WT
FAX1-2
FAX1-2
FAX1-1
FAX1-1
ABCA9-2
ABCA9-1
ABCA9-2
ABCA9-1
FAX1/ABCA9-2
FAX1/ABCA9-1
FAX1/ABCA9-2
FAX1/ABCA9-1
Additional file 4: Figure S4. Effect of AtFAX1- and AtABCA9-OEs on vegetative tissues. a Morphology of 3-week-old plants. Bar=5 cm. b The fresh weight and leaf number of 3-week-old overground plants. Values are means ± SE with three batch of repeats, each repeat with 12 plants of each line. Capital letters indicate a significant difference (P < 0.01) based on Duncan-test.
