# Supplementary material for: Increased expression of fatty acid and ABC transporters enhances seed oil production in camelina
Source: Biotechnol Biofuels. 2021 Feb 27;14:49. doi: 10.1186/s13068-021-01899-w (PMC7913393; doi:10.1186/s13068-021-01899-w)
Supplement: Supplementary file 6 — Additional file 6: Figure S6. Alterations of PC, PE, PG, MGDG, and DGDG species (mol%) in developing pods of AtFAX1- and AtABCA9-OEs. [file 13068_2021_1899_MOESM6_ESM.pptx]

## Slide 1
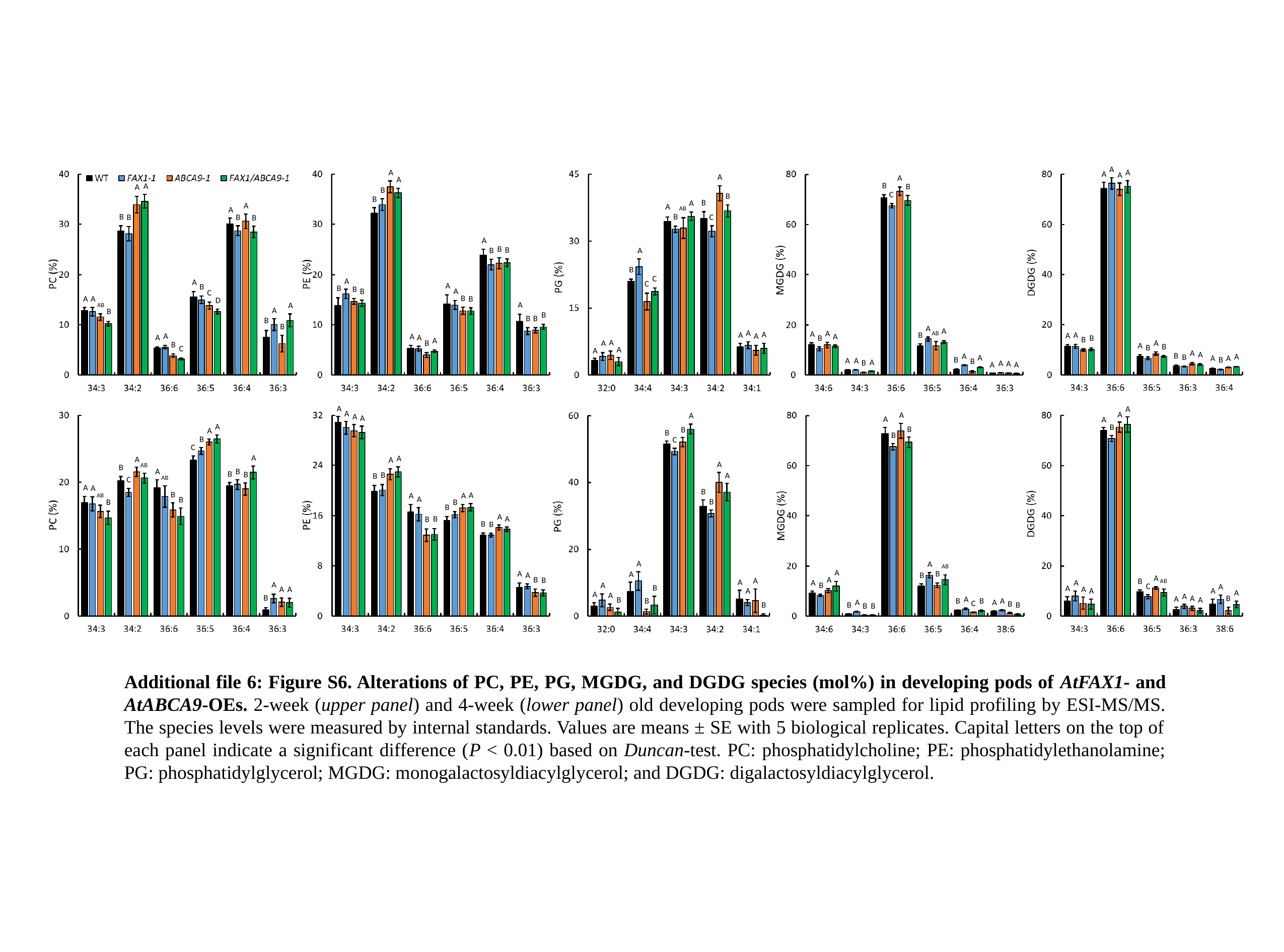

A
A
A
A
A
A
A
A
B
A
B
A
B
C
B
B
B
A
A
A
A
AB
B
B
C
B
B
B
A
B
B
B
A
B
C
A
A
C
A
B
B
B
A
B
C
B
B
A
A
D
A
A
AB
A
B
B
B
B
B
B
A
A
A
A
A
A
AB
B
A
A
A
A
A
A
A
A
A
B
B
B
A
A
A
A
B
B
A
B
B
C
A
A
A
A
B
A
A
B
A
A
A
B
B
A
A
B
A
B
A
A
A
A
A
A
A
A
A
A
A
A
A
A
A
B
B
B
A
B
B
B
C
C
A
A
A
A
A
AB
B
B
A
B
B
B
A
B
AB
C
A
A
B
B
A
A
A
AB
A
B
B
B
B
B
A
A
B
B
B
B
A
A
AB
A
A
B
A
A
B
A
A
B
B
A
B
A
AB
A
A
A
B
A
C
A
B
A
A
A
A
A
A
A
A
A
A
A
B
A
B
A
A
B
A
B
B
B
A
A
A
C
B
B
B
B
B
B
Additional file 6: Figure S6. Alterations of PC, PE, PG, MGDG, and DGDG species (mol%) in developing pods of AtFAX1- and AtABCA9-OEs. 2-week (upper panel) and 4-week (lower panel) old developing pods were sampled for lipid profiling by ESI-MS/MS. The species levels were measured by internal standards. Values are means ± SE with 5 biological replicates. Capital letters on the top of each panel indicate a significant difference (P < 0.01) based on Duncan-test. PC: phosphatidylcholine; PE: phosphatidylethanolamine; PG: phosphatidylglycerol; MGDG: monogalactosyldiacylglycerol; and DGDG: digalactosyldiacylglycerol.
