# Supplementary material for: Increased expression of fatty acid and ABC transporters enhances seed oil production in camelina
Source: Biotechnol Biofuels. 2021 Feb 27;14:49. doi: 10.1186/s13068-021-01899-w (PMC7913393; doi:10.1186/s13068-021-01899-w)
Supplement: Supplementary file 7 — Additional file 7: Figure S7. Gene expression level of AtFAX1 and AtABCA9 in developing seeds of camelina OE lines. [file 13068_2021_1899_MOESM7_ESM.pptx]

## Slide 1
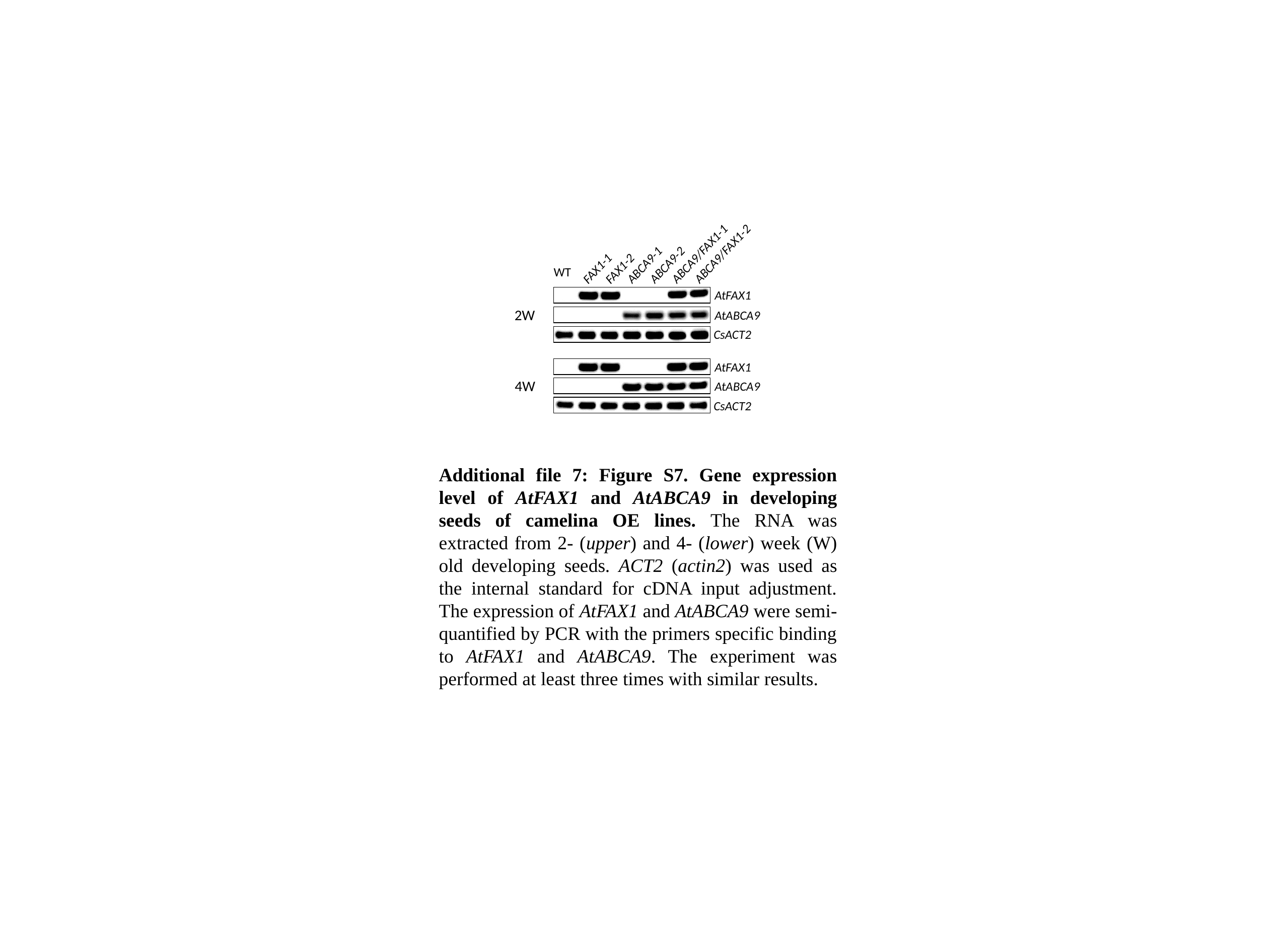

ABCA9/FAX1-1
ABCA9/FAX1-2
ABCA9-2
ABCA9-1
FAX1-1
FAX1-2
WT
AtFAX1
2W
AtABCA9
CsACT2
AtFAX1
4W
AtABCA9
CsACT2
Additional file 7: Figure S7. Gene expression level of AtFAX1 and AtABCA9 in developing seeds of camelina OE lines. The RNA was extracted from 2- (upper) and 4- (lower) week (W) old developing seeds. ACT2 (actin2) was used as the internal standard for cDNA input adjustment. The expression of AtFAX1 and AtABCA9 were semi-quantified by PCR with the primers specific binding to AtFAX1 and AtABCA9. The experiment was performed at least three times with similar results.
